# Red blood cell-tumor cell interactions promote tumor cell progression

**DOI:** 10.1186/s13046-025-03376-w

**Published:** 2025-04-29

**Authors:** Thais Pereira-Veiga, Celso Yáñez-Gómez, Aleksi Pekkarinen, Carmen Abuín, Christine Blechner, Miriam González-Conde, Christian Mess, Sabine Vidal-y-Sy, Ayham Moustafa, Bente Siebels, Ana B. Dávila-Ibáñez, Pablo Cabezas-Sainz, Maider Santos, Laura Sánchez, Joao Gorgulho, Julian Götze, Kira Meißner, Juan Cueva, Patricia Palacios, Alexandra Cortegoso, Teresa Curiel, Carmela Rodríguez, Marta Carmona, Luis León-Mateos, Alicia Abalo, Laura Muinelo-Romay, Sven Peine, Milena Schmidt, Nadine Heuer-Olewinski, Martin Reck, Mustafa Abdo, Katrin Lamszus, Alexander T. Bauer, Rafael López-López, Klaus Pantel, Sabine Windhorst, Harriet Wikman, Clotilde Costa

**Affiliations:** 1 Department of Tumor Biology, University Medical Center Hamburg-Eppendorf, Hamburg, Germany; 2 Translational Medical Oncology Group, Health Research Institute of Santiago de Compostela (IDIS), Oncomet, Santiago de Compostela, Spain; 3 Department of Biochemistry and Signal Transduction, University Medical Center Hamburg-Eppendorf, Hamburg, Germany; 4 Department of Dermatology and Venereology, University Medical Center Hamburg-Eppendorf, Hamburg, Germany; 5 Section Mass Spectrometry and Proteomics, University Medical Center Hamburg-Eppendorf, Hamburg, Germany; 6 CIBERONC, Centro de Investigación Biomédica en Red Cáncer, Madrid, Spain; 7 Zoology, Genetics and Physical Anthropology Department, Universidade de Santiago de Compostela, Campus de Lugo, Lugo, Spain; 8 Department of Oncology, Hematology and Bone Marrow Transplantation with Section of Pneumology, University Medical Center Hamburg-Eppendorf, Hamburg, Germany; 9 University Clinical Hospital of Santiago (CHUS/SERGAS), Santiago de Compostela, Spain; 10 Liquid Biopsy Analysis Unit, Oncomet, Santiago de Compostela, Spain; 11 Institute of Transfusion Medicine, University Medical Center Hamburg-Eppendorf, Hamburg, Germany; 12 Airway Research Center North, LungenClinic Grosshansdorf, German Center for Lung Research, Grosshansdorf, Germany; 13 Department of Neurosurgery, University Medical Center Hamburg-Eppendorf, Martinistr. 52, 20246 Hamburg, Germany

**Keywords:** Red blood cells (RBCs), Breast cancer, Lung cancer, Circulating tumor cells, Liquid biopsy, Actin remodeling

## Abstract

A critical step in the metastatic cascade is the survival of circulating tumor cells (CTCs) within the bloodstream. Although interactions between CTCs and various hematopoietic cells have been described, the role of red blood cells (RBCs) remains underexplored. This study investigated the interactions between tumor cells and RBCs from breast and lung cancer patients, revealing significant phenotypic and functional changes in tumor cells, unlike interactions with RBCs from healthy donors. Tumor cell and patient-derived RBC co-cultures increased tumor cell attachment and induced morphological changes. RBC-primed tumor cells showed increased adhesion, disruption of the endothelial barrier, and invasiveness, both in vitro and in vivo. Global proteome changes, including actin remodeling and VASP accumulation at cell edges, promote directional migration. RBCs from patients with metastatic breast cancer also upregulate PAK4, enhancing migration and epithelial-mesenchymal transition, whereas PAK4 inhibition reduces these effects. Clinically, a higher red blood cell distribution width (RDW) in patients with metastasis is associated with increased CTC counts and poor outcomes. This study highlights the previously unrecognized role of RBCs in promoting metastatic behavior in cancer cells and suggests potential therapeutic targets, such as PAK4, to counteract these effects.

**Supplementary Information:**

The online version contains supplementary material available at 10.1186/s13046-025-03376-w.

## Background

Metastasis is the leading cause of cancer-related deaths [1]. Although extensive efforts have been made to investigate the metastatic cascade, significant gaps remain in our understanding of the entire process. The metastatic cascade is an intricate sequence of events in which cancer cells move from the primary tumor to other locations within the body. It begins with local invasion, followed by the cancer cells entering nearby tissues and blood vessels. Subsequently, these cells intravasate into the bloodstream or lymphatic system, becoming circulating tumor cells (CTCs), and travel to distant organs. In the blood, CTCs encounter significant challenges related to their survival in circulation and their ability to extravasate to new metastatic sites [2]. Numerous important interactions between CTCs and blood components, such as platelets, neutrophils, monocytes, and endothelial cells, are crucial in this process. Many of these interactions are permissive or necessary for CTC survival in the bloodstream [2]. Surprisingly, the role of the most abundant component of blood, red blood cells (RBCs), remains almost unaddressed. RBCs, initially thought to only carry oxygen, are now recognized for their role in maintaining metabolic homeostasis and influencing cellular processes [3]. Furthermore, RBCs have been implicated in the pathophysiology of diseases such as diabetes, Alzheimer’s disease, multiple sclerosis, and rheumatoid arthritis [4, 5, 6, 7].

While the interaction between tumor cells and RBCs remains understudied, the literature suggests that RBCs can interact with and modulate other immune cells, such as eosinophils and lymphocytes [8, 9, 10]. RBCs have membrane-bound proteoglycans and glycoproteins that bind cytokines, thereby modulating inflammatory processes and potentially triggering cytokine storms, such as those occurring after blood transfusions [11, 12]. Cytokine profile alterations and immune modulation in RBCs have also been observed upon exposure to cancer cells [12]. Moreover, studies using mouse models have demonstrated that RBCs and hemoglobin can act as endogenous danger signals, promoting the proliferation of breast and melanoma tumor cells, as well as recruiting and polarizing macrophages [13]. Additionally, Helwa et al. proposed that tumor cells may interact with RBCs, potentially through galectin-4, which is primarily expressed in gastrointestinal epithelial cells and has implications in various cancers [14]. Furthermore, we have recently observed that the presence of RBCs in CTCs short-term cultures is associated with poorer patient outcomes [15], and we have also demonstrated that the protein profiles of RBCs in cancer patients are altered compared to those in cancer-free donors [16].

Clinically, research has focused on the association between red blood cell distribution width (RDW) and cancer. RDW, a routinely measured parameter in a complete blood count, is often altered in cancer patients and has been proposed as a novel biomarker for the diagnosis and prognosis of different tumor types [17, 18, 19]. However, the biology underlying this phenomenon remains poorly understood.

CTCs have the opportunity to interact directly with numerous RBCs in the bloodstream. To explore the possible consequences of these interactions, we conducted both in vitro and in vivo experiments using breast and lung cancer cell lines as models. RBCs from breast and lung cancer patients were used to prime tumor cells for these experiments. This study is the first to comprehensively investigate the potential connections and biological consequences of interactions between tumor cells and RBCs.

## Results

### RBCs from metastatic patients interact with tumor cells, causing their phenotypic transformation

**Fig. 1:**
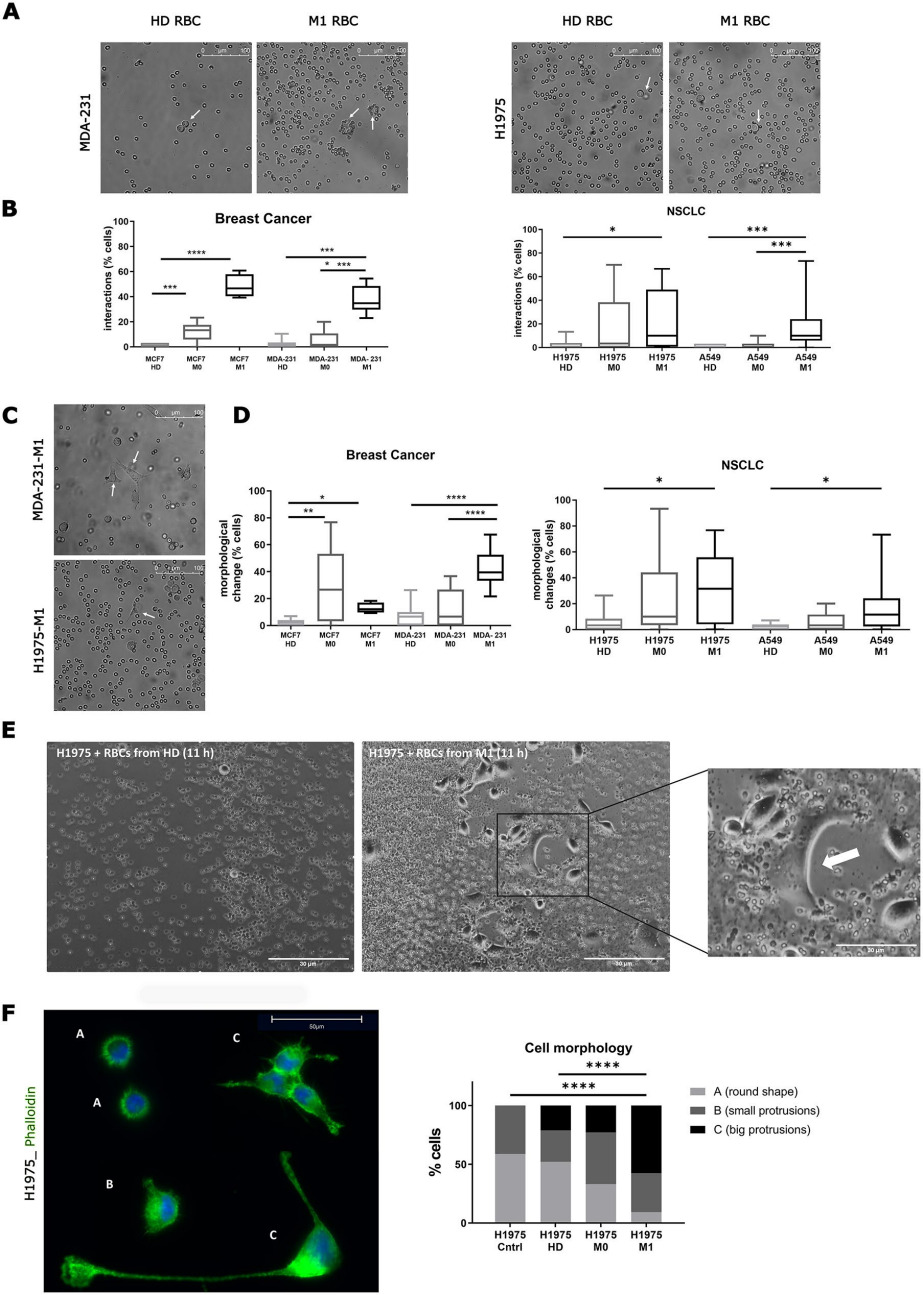
RBCs and tumor cells interact in suspension. (**A**) Exemplary pictures depicting the interaction between RBCs (from healthy donors (HD) and metastatic patients (M1)) and tumor cells (MDA-MB-231 and H1975), showing a ring of RBCs from M1 patients attaching to a tumor cell, or no interaction for RBCs from HD (marked with a white arrow). (**B**) Boxplots showing the percentage of tumor cells (MCF7, MDA-MB-231, H1975, and A549) interacting with RBCs from HD, M0 or M1 patients (*n* = 15 per group, except MCF7 M1: *n* = 4, 30 cells quantified/sample, triplicates). (**C**) Exemplary pictures depicting the change of morphology and adhesion of tumor cells (MDA-MB-231 and H1975) under ultra-low attachment conditions. (**D**) Percentage of cells showing changes in morphology when MCF7, MDA-MB-231, H1975 or A549 are co-cultured with RBCs from M0 or M1 cancer patients compared to HD (*n* = 10–15 per group, except MCF7 M1: *n* = 4, 30 cells quantified/sample, triplicates). (**E**) Representative images after 11 h of co-culture with H1975 and RBCs from either a HD or a M1 NSCLC patient from the time-lapse. The white arrow indicates the formation of lamellipodia-like structures. (**F**) Representative immunofluorescence images of morphological changes visualized by Alexa-fluor488-phalloidin staining of H1975 cells primed with RBCs (left panel) and quantification of type of morphological changes (right panel) (*n* = 5 per group, triplicates). * *P* < 0.05, ** *P* < 0.01, *** *P* < 0.001

To evaluate the interaction of RBCs with tumor cells in vitro, we co-cultured breast cancer (MDA-MB-231 and MCF-7) and lung cancer (H1975, A549) cell lines in suspension for 24 h with RBCs isolated from patients with localized (M0) or metastatic (M1) breast cancer or non-small cell lung cancer (NSCLC), as well as from healthy donors (HD). We observed that M1 RBCs interacted more frequently with tumor cells, whereas HD RBCs did not (Fig. 1A). Thus, we observed a significantly higher percentage of cancer patients whose RBCs interacted with tumor cells compared to HD patients (Fig. S1A). Next, we quantified the interaction between individual tumor cells and RBCs from cancer patients and HD. Highly significant differences were found in the interaction between tumor cells and M1 RBCs compared with HD RBCs (Fig. 1B).

After co-culturing tumor cell lines with M1 RBCs in suspension, the tumor cells were significantly more frequently attached to the bottom of the plates and exhibited morphological changes compared to those primed with HD RBCs (Fig. 1C-D). This change was characterized by transitioning from a rounded shape to an adherent growth form, exhibiting protrusions. We conducted a 22-hour in vitro time-lapse live-cell imaging assay of H1975 cells with M1 or HD RBCs under ultra-low attachment conditions to further investigate this observation. Consistent with our previous data, H1975 cells exhibited significant phenotypic changes after 11 h when co-cultured with M1 RBCs, but not when co-cultured with HD RBCs. Representative images from time-lapse live-cell imaging are shown in Fig. 1E (complete video available in Video S1, S2).

Based on the previous observation that cells co-cultured with M1 RBCs form long protrusions, phalloidin staining was performed to visualize actin structures and identify different cell morphologies. To this end, H1975 cells were primed with HD, M0, or M1 RBCs under adherent conditions. Tumor cells with large protrusions were significantly more frequently observed in cells primed with M1 RBCs. In contrast, round shapes without protrusions were more common in cells primed with HD RBCs (Fig. 1F). This indicates that priming tumor cell lines with RBCs from metastatic patients induces significant actin rearrangement, favoring the formation of cells with numerous large protrusions.

### Patient-derived RBCs promote tumor cell metastatic potential

Considering the significant impact of M1 RBCs on tumor cell phenotype, our next goal was to investigate whether priming human cancer cell lines with RBCs affects their functional abilities, including migration, adhesion, proliferation, and invasion—key steps in metastasis.

**Fig. 2:**
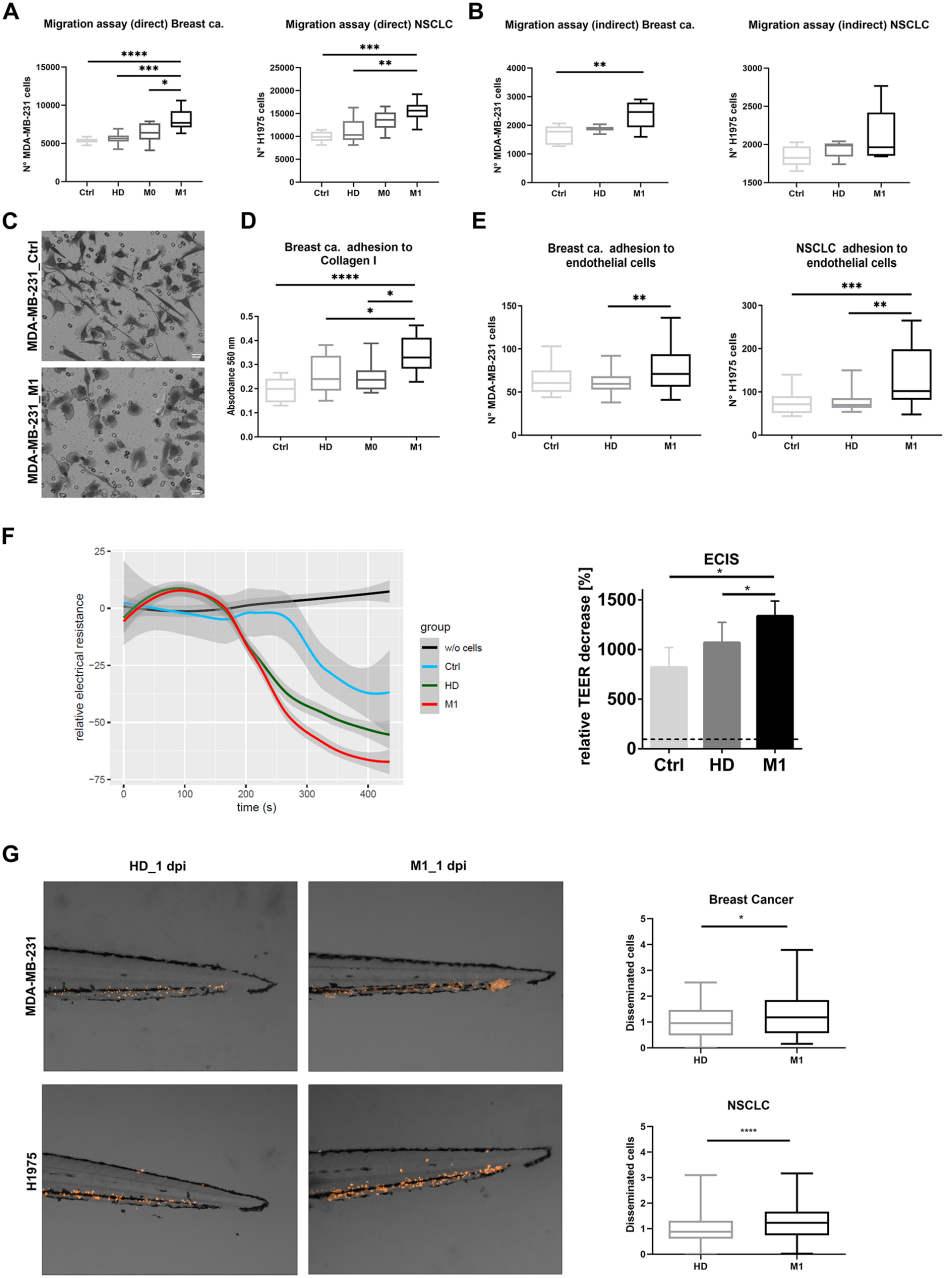
Functional evaluation of tumor cells after RBCs contact. (**A**) Boxplots showing tumor cell transwell migration following priming (direct interaction) with RBCs from healthy donors (HD), M0, or M1 patients for breast cancer (MDA-MB-231, left panel) and non-small cell lung cancer (NSCLC) cells (H1975, right panel), or non-primed control (Ctrl) cells (n = 5 per group, triplicates). (**B**) Boxplot illustrating tumor cell transwell migration when RBCs from HD or M1 are placed in the bottom well (indirect interaction) for MDA-MB-231 (left panel) and H1975 (right panel), compared to non-primed control cells (Ctrl) (n = 5 per group, triplicates). (**C**) Representative images of MDA-MB-231 cells observed in the migrated fraction from the indirect transwell assay, showing cells without RBC priming (Ctrl) in the upper panel and cells primed with M1 RBCs in the bottom panel, with the latter displaying notable morphological changes (**D**) Absorbance values reflecting the adhesion to collagen I by MDA-MB-231 after priming with RBCs from HD, M0 or M1 (n= 5 per group, triplicates). A negative control condition without priming with RBCs was included (Ctrl, n= 5). (**E**) Boxplots representing the MDA-MB-231 and H1975 cell counts (primed with RBCs from HD, M1 or non-primed tumor cells (n=5 per group, triplicates)) that adhered to endothelial cells (HUVEC). (**F**) Electric Cell-substrate Impedance Sensing (ECIS) assay results indicate a greater disruption of the endothelial barrier function when H1975 cells are primed with RBCs from M1 NSCLC patients (red line) compared to HD (green line), unprimed H1975 cells (blue line), or medium without cells (black line) (n=3 per group, duplicates)(left panel). Boxplot of the normalized transendothelial resistance (TER) of H1975 cells primed with HD or M1 RBCs and unprimed cells (Ctrl) (right panel). (**G**) Representative images of tumor cell dissemination in the tail region of the zebrafish embryos injected with either MDA-MB-231 or H1975 cancer cell lines primed with RBCs from metastatic patients or HD, at 1 day post injection (dpi) (ntotal = 75 embryos per group, triplicates; survival MDA-MB-231 HD = 94, M1 = 75; survival H1975 HD = 162, M1 = 176). The main images are a superposition of a fluorescence image and a bright field image of the same embryo. Quantification of disseminated tumor cells on the zebrafish embryo's tails at 1dpi. * *P* < 0.05, ** *P* < 0.01, *** *P* < 0.001

First, we studied whether RBCs from patients with metastasis could affect tumor cell proliferation and migration. MDA-MB-231 and H1975 cells were primed with M1, M0, or HD RBCs. No significant differences were found in the proliferation rates among the different conditions (Fig. S1B), but both cell lines showed significantly increased migration capability after 24 h of priming with M1 RBCs compared to HD (Fig. 2A and Fig. S1C). Next, we evaluated whether direct contact between tumor cells and RBCs was necessary to enhance the migratory capacity of tumor cells. RBCs were seeded on the lower part of a transwell plate, preventing direct contact with tumor cells. In MDA-MB-231 cells, RBCs from metastatic breast cancer patients showed migration similar to the direct contact setting and higher migration than non-primed cells. However, in H1975 cells, although there was a trend toward higher migration on metastatic RBCs, no significant difference was observed (Fig. 2B). Notably, a migratory phenotype characterized by membrane protrusions resembling lamellipodia was detected, mainly in the presence of M1 RBCs (Fig. 2C).

Based on the observed tumor cell adhesion on ultra-low attachment surfaces, we assessed the adhesion of MDA-MB-231 cells to a collagen I matrix after priming with RBCs. As shown in Fig. 2D, priming with M1 RBCs significantly increased adhesion compared to HD. Similarly, we found that priming both MDA-MB-231 and H1975 cells with M1 RBCs significantly enhanced their adhesion to endothelial cells (HUVECs) in vitro (Fig. 2E). Further analyses using the Electric Cell-substrate Impedance Sensing (ECIS) assay were performed to assess endothelial barrier function in the presence of tumor cells primed with M1 or M0 RBCs or unprimed cells. This experiment revealed a 25% increase in the disruption of endothelial barrier function induced by H1975 cells primed with M1 RBCs compared to HD RBCs (Fig. 2F).

To further validate our findings in vivo, zebrafish experiments were performed. MDA-MB-231 and H1975 cells primed with M1 or HD RBCs were injected into the Duct of Cuvier in zebrafish embryos. Tumor cells primed with M1 RBCs showed significant enhancement of dissemination to the caudal region (Fig. 2G).

### Metastatic RBCs priming induces large gene expression changes and enhances breast tumor cell migration and adhesion via *PAK4*

**Fig. 3:**
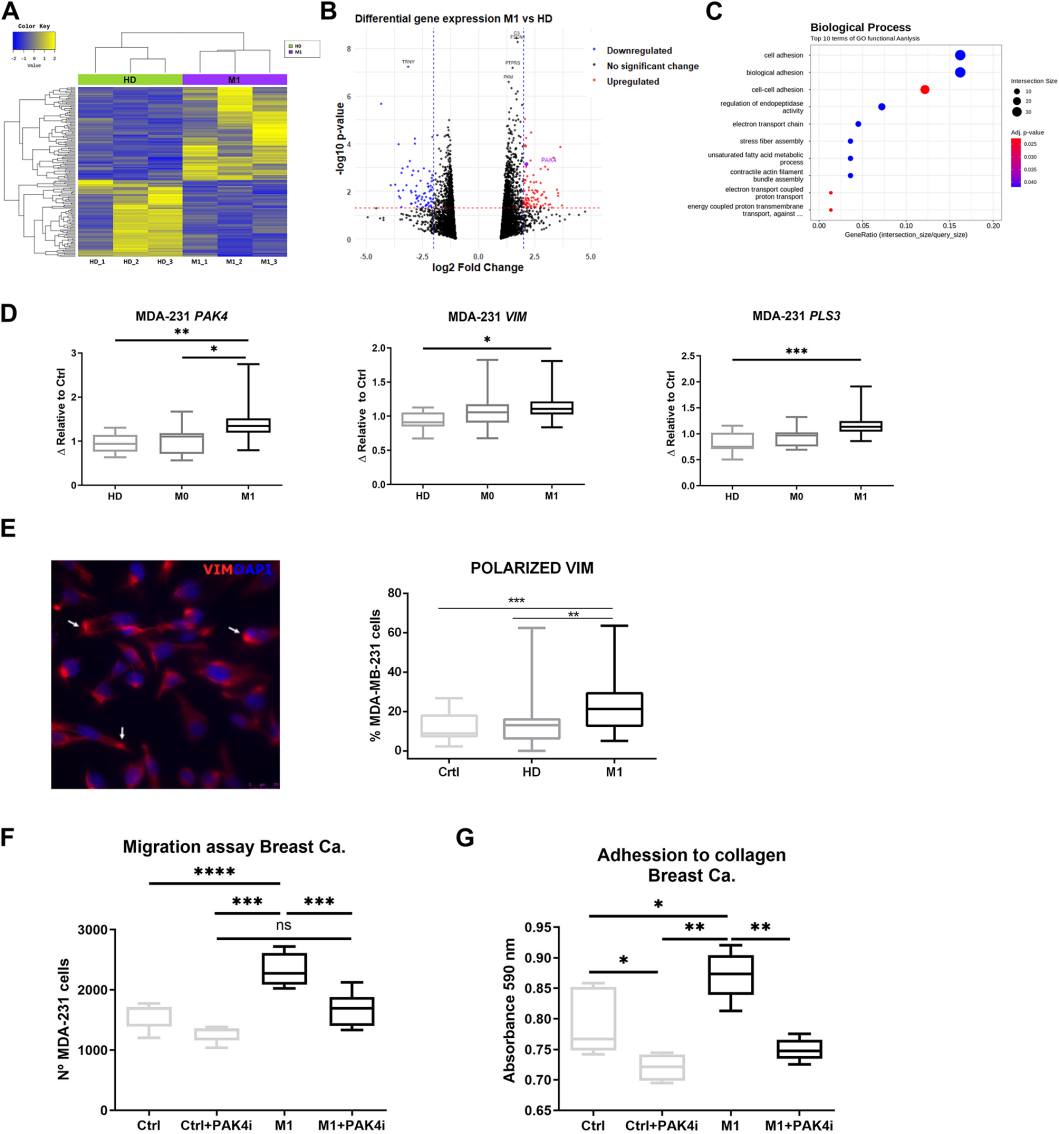
Transcriptomic and functional analysis of MDA-MB-231 cells primed with RBCs or non-primed. (**A**) Heatmap showing results of the hierarchical clustering analysis of the significantly differentially expressed genes in MDA-MB-231 primed with RBCs from either HD (green) or M1 patients (purple) (n= 3 per group). (**B**) Volcano plot for the differentially expressed genes between HD and M1 samples. (**C**) Gene ontology analysis of the differentially expressed genes displaying the biological processes altered on MDA-MB-231 after co-cultivation with RBCs. (**D**) PAK4, VIM and PLS3 gene expression was analyzed by RT-qPCR, from MDA-MB-231 samples co-cultured with HD, M0 or M1 RBCs. Samples were relativized to B2M and normalized to Δct from negative control (non-primed cells, Ctrl) (n= 14 per group, triplicates). (**E**) A representative immunofluorescence image (left panel) highlights vimentin expression (in red) in MDA-MB-231 cells primed with M1 RBCs, with polarized regions marked by white arrows. Boxplot showing the percentage of polarized MDA-MB-231 cells in each group: Crtl(n=4) or after priming with RBCs from HD and M1 patients (n=6, each) (right panel). (**F**) Graph representing migration of MDA-MB-231 co-cultured with metastatic breast cancer RBCs or without RBCs in the presence or absence of PAK4 inhibitor (PAKi, LCH-7749944) (n= 5 per group, duplicates). (**G**) Graph representing adhesion to collagen I of MDA-MB-231 primed with metastatic breast cancer RBCs or without RBCs in the presence or absence of PAK4i (n= 5 per group, triplicates). * *P* < 0.05, ** *P* < 0.01, *** *P* < 0.001

Based on the previous findings regarding the impact of patient-derived RBCs on tumor cell phenotype and behavior, we investigated their effect on the gene expression profiles of tumor cells. Whole-transcriptome sequencing revealed significant alterations in gene expression profiles between MDA-MB-231 cells primed with HD or M1 RBCs (Fig. 3A-B) and with non-primed cells or liposomes as controls (Fig. S1D). In total, 91 genes were significantly upregulated, while 75 genes were downregulated in cell lines primed with either M1 or HD RBCs. Gene Ontology analysis highlighted substantial changes in adhesion-related biological processes and genes involved in actin filament contraction and assembly (Fig. 3C).

Among the genes with higher expression in the metastatic group (Table S4), *PAK4* was selected due to its role in survival, migration, and epithelial-mesenchymal transition (EMT). The changes in expression were validated by RT-qPCR in tumor cells primed with a larger cohort of RBCs from cancer patients and HD. *PAK4* was overexpressed in MDA-MB-231 cells primed with M1 RBCs compared to cells primed with HD or M0 RBCs (Fig. 3D). Further analysis of EMT-related genes in MDA-MB-231 cells revealed significantly higher levels of vimentin (*VIM*) and plastin 3 (*PLS3*) in cells co-cultured with M1 RBCs compared to those primed with HD RBCs (Fig. 3D).

Immunofluorescence was performed on MDA-MB-231 cells, with and without RBC priming, to assess vimentin and plastin3 expression. The results showed distinct vimentin polarization in the M1 primed group compared to non-primed cells or those primed with HD RBCs. Tumor cells primed with HD RBCs showed no significant statistical differences compared to control cells. Plastin3 showed a tendency toward increased expression in M1-primed MDA-MB-231 cells (Fig. S1E).

To further explore the role of PAK4 in the functional changes observed in MDA-MB-231 cells, we used a PAK4-specific inhibitor (LCH-7749944, PAK4i) (Fig. S1F). PAK4 inhibition significantly reduced the migration capacity of MDA-MB-231 cells primed with M1 RBCs and untreated cells. Importantly, no significant differences were observed between non-primed cells and M1 RBC-primed cells treated with PAK4i, indicating that the inhibitor reversed the enhanced migratory capacity of these cells (Fig. 3F). Consistent with these findings, PAK4 inhibition in MDA-MB-231 cells primed with M1 RBCs reduced adhesion to collagen I to levels comparable to those in non-primed cells (Fig. 3G).

### RBCs from metastatic cancer patients induce global proteomic changes including actin remodeling in lung cancer cells

**Fig. 4:**
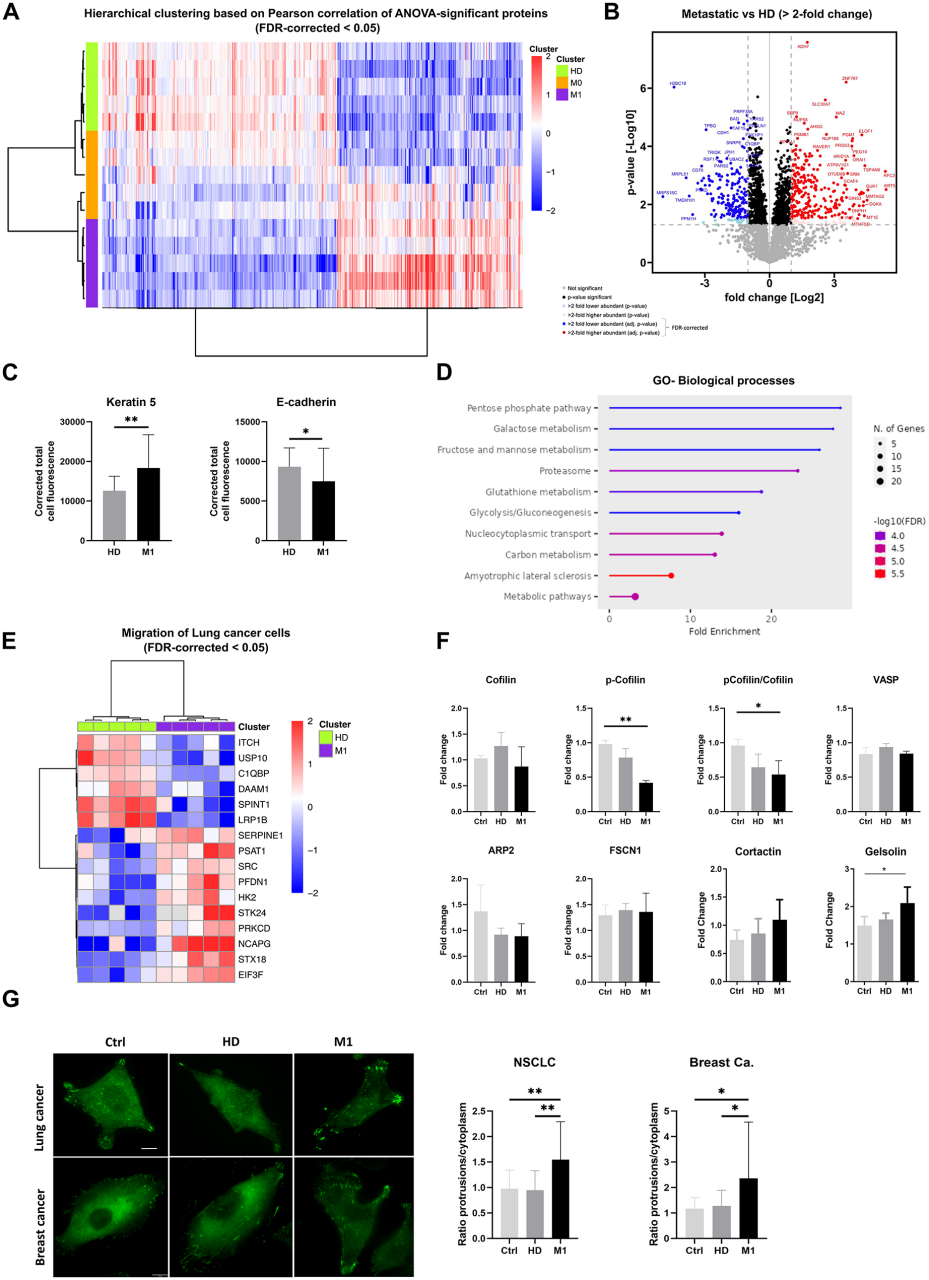
Proteomic analysis of tumor cells primed with RBCs from cancer patients, HD and non-primed (Ctrl). (**A**) Hierarchical clustering based on Pearson correlation of ANOVA-significant proteins of H1975 cell primed with RBCs from HD, M0 and M1 patients (*n* = 5 per group, quadruplicates). (**B**) Volcano plot for the differentially abundant proteins between M1 and HD samples. (**C**) Bar plot representing the quantification of the fluorescence intensity in H1975 cells stained with anti-K5 and anti-E-cadherin, post-priming with RBCs from HD or metastatic patients (*n* = 30 cells quantified/group, *n* = 3). (**D**) Gene ontology analysis of the differentially expressed proteins showing the biological processes affected on H1975 cells after priming with RBCs. (**E**) Hierarchical clustering of the differential abundance of the proteins from the dataset involved in migration of lung cancer cell lines, between H1975 cells primed either with HD or M1 RBCs (FDR < 0.05, Fold-change > 2). (**F**) Barplots representing the quantification of western blots for the proteins Cofilin, p-Cofilin, VASP, Arp2, FSCN1, Cortactin and Gelsoline in H1975 primed with RBCs from HD or metastatic patients as well as non-primed cells (Ctrl) (*n* = 3–4 per group). (**G**) Representative images of H1975 cells and MDA-MB-231 cells, stained for the protein VASP after priming with RBCs. The white arrows indicate the accumulation of VASP on the cellular protrusions (left panel). Quantification of the ratio of VASP-fluorescence intensity in protrusions/cytoplasm on H1975 and MDA-MB-231 cells stained with VASP after being primed with RBCs from HD or metastatic RBCs, as well as non-primed cells (*n* = 30 cells quantified/group, *n* = 3) (right panel). * *P* < 0.05, ** *P* < 0.01, *** *P* < 0.001

To identify the underlying processes and molecular targets influenced by RBCs, we investigated the global proteomic changes in H1975 cells primed with RBCs from NSCLC patients. In total, 4,414 proteins were identified, of which 626 showed significant differential abundance between cells primed with HD, M0, and M1 RBCs (Fig. 4A-B, Fig. S2A). These findings indicate substantial remodeling of the tumor cell proteome following contact with RBCs. Specifically, we observed 269 proteins with increased abundance and 176 with decreased abundance in H1975 cells primed with M1 RBCs compared to those primed with HD RBCs. Among the top 20 differentially abundant proteins, two EMT-related proteins, Keratin 5 and E-cadherin, were identified (Table S5), and their expression was further validated by immunofluorescence staining (Fig. 4C).

Gene ontology analysis revealed significant alterations in the enrichment of proteins related to pathways primarily involved in metabolic processes (Fig. 4D). Further analysis using IPA and z-score algorithms highlighted migration, cell movement, invasion, and DNA repair as significantly increased, while autophagy was predicted to decrease. Specifically, the migration of lung cancer cell lines was predicted to increase, with 16 proteins showing significant differential expression (Fig. 4E, Fig. S2B-C). Additionally, 8 upstream regulatory proteins were predicted to be activated, including Rab-like protein 6 (RABL6), heat shock factor 1 (HSF1), epidermal growth factor (EGF), and ERBB2, all of which are involved in the progression of various cancers (Fig. S2D).

Given the crucial role of cytoskeletal dynamics in regulating cancer cell adhesion, migration, and invasion, we examined the activity and expression levels of proteins involved in actin remodeling in more detail. We first analyzed the activity of Cofilin-1, an actin-severing protein and downstream effector of the Cdc42-PAK-LIMK pathway, which is inhibited by LIMK-mediated phosphorylation at Ser3. The level of phosphorylated cofilin (p-cofilin) was significantly decreased in cancer cells treated with M1 RBCs, while the total Cofilin-1 protein level remained unchanged, resulting in a significantly decreased p-cofilin/cofilin-1 ratio (Fig. 4F, Fig. S2E).

Next, to assess whether proteins involved in F-actin elongation or bundling were altered in cells treated with RBCs from metastatic patients, we analyzed the protein levels of VASP, Arp2, Fascin 1, Cortactin, and Gelsolin by Western blotting. Gelsolin showed significantly higher expression after priming with M1 RBCs, while no significant differences in the total levels of these other proteins were observed between the control group and cells incubated with M1 RBCs, but a trend in Cortactin (Fig. 4F, Fig. S2F). Although no differences were observed in total VASP protein expression, immunofluorescence analysis revealed a significant increase of VASP at the protrusions of both lung and breast cancer cells (Fig. 4G). This suggests that signals from RBCs of metastatic patients lead to the accumulation of VASP at the leading edge of cancer cells, promoting directed migratory capabilities.

### Clinical implications of the interaction of RBCs from metastatic patients with CTCs

**Fig. 5:**
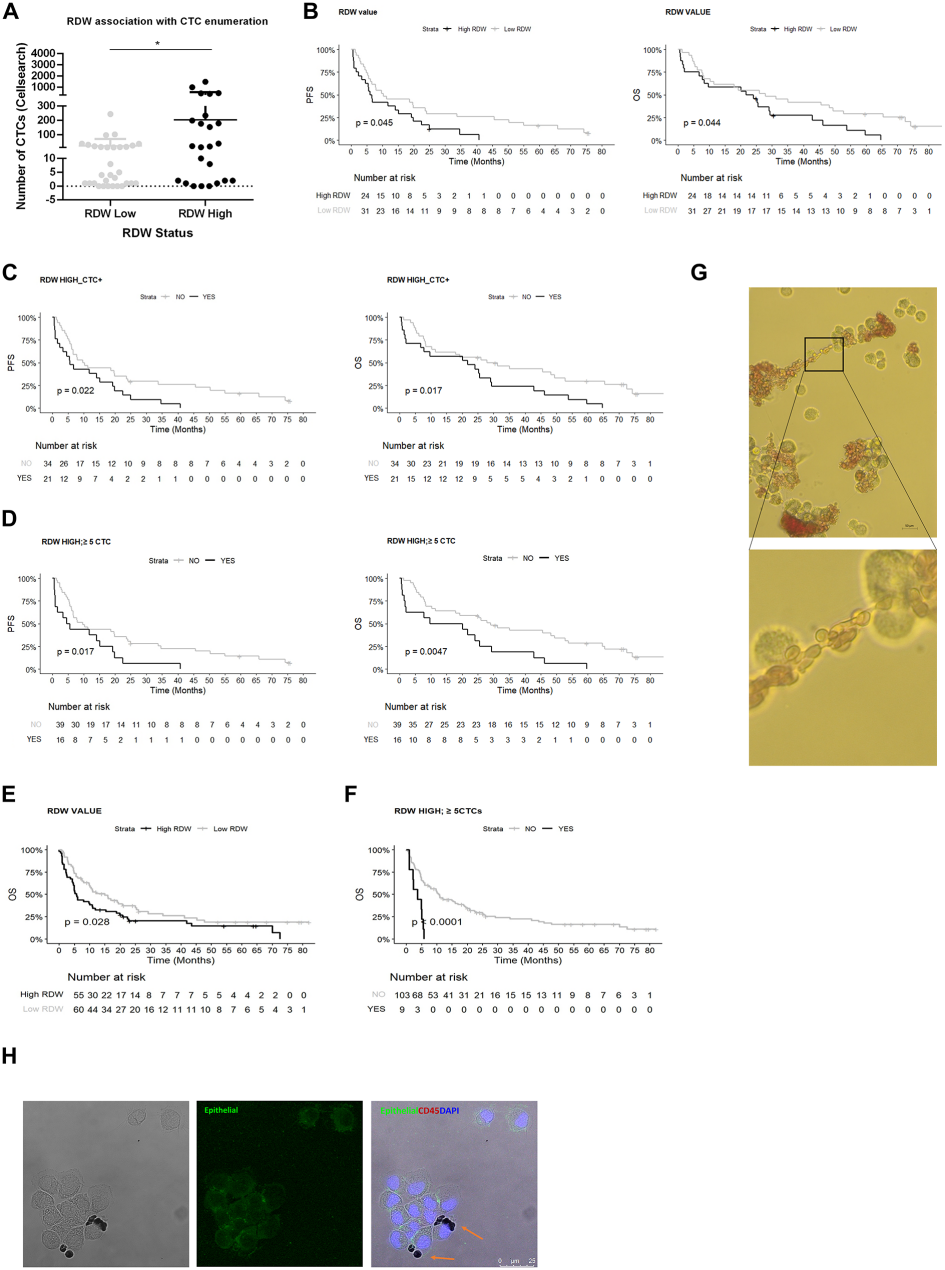
RBC interaction with CTCs. (A) Boxplot showing the association between RDW status and CTC count, determined by CellSearch® from 7.5 mL blood of metastatic breast cancer patients (n= 55). (B) Kaplan–Meier plots of PFS and OS for high (black) or low (grey) RDW values (cut-off = 14.5) in breast cancer patients (median PFS: 6.38 vs 10.29 months, P = 0.04; median OS: 22.9 vs 27.8 months, P = 0.04) (C) Kaplan–Meier plots of PFS and OS for patients with combined high RDW and ≥ 1 CTC (RDW HIGH_CTC+, black) or not (grey) (RDW cut-off = 14.5) (median PFS: 5.56 vs 10.01 months, P = 0.022; median OS: 21.8 vs 27.8 months, P = 0.017) (D) Kaplan–Meier plots of PFS and OS for breast cancer patients with a combination of high RDW and ≥ 5 CTC (RDW HIGH_≥ 5 CTC, black) or not (grey) (RDW cut-off = 14.5) (median PFS: 9.73 vs 5.08 months, P = 0.017; median OS: 29.1 vs 14.9 months, P = 0.0047) (E) Kaplan–Meier plot of OS for high (black) or low (grey) RDW values (cut-off = 14.5) in NSCLC patients (median OS: 5.7 vs 15 months, P = 0.028) (F) Kaplan–Meier plot of OS for NSCLC patients with a combination of high RDW and ≥ 5 CTC (RDW HIGH_≥ 5 CTC, black) or not (grey) (RDW cut-off = 14.5) (median OS: 3.65 vs 10.81 months, P = 0.00009) (G) Representative bright field microscope images of CTCs from a metastatic breast cancer patient in ex vivo culture, with attached RBCs. (H) Representative confocal microscopy images of immunofluorescence characterization of CTCs from a metastatic breast cancer patient after 10 days of culture. Staining used anti-human epithelial markers (EpCAM, E-cadherin, PanCK, green), anti-CD45 (red), and DAPI (blue). RBCs attached to CTCs are indicated with orange arrows. *P < 0.05

To assess whether alterations in patients’ RBCs have clinical implications, we investigated whether CTC counts correlate with RDW values and clinical outcomes in both breast and NSCLC patients. A total of 7.5 ml of blood from 55 metastatic breast cancer patients was analyzed for CTC enumeration using the CellSearch^®^ system at the time of metastatic disease diagnosis (prior to starting therapy). The mean number of CTCs was 101.8. Notably, 84.0% of patients had at least one CTC, with 51.4% meeting the standard cut-off of ≥ 5 CTCs/7.5 ml of blood [20]. Patients with a high RDW (≥ 14.5) had significantly higher CTC counts compared to those with a normal RDW (Fig. 5A).

We then performed survival analysis based on RDW levels and found that a high RDW was associated with poorer progression-free survival (PFS) and overall survival (OS) (Fig. 5B). Interestingly, the presence of ≥ 1 CTC was linked to worse OS, but not PFS (Fig. S3A-D). The combination of high RDW with either ≥ 1 CTC or ≥ 5 CTCs was associated with worse PFS and OS, showing greater significance than RDW or CTCs alone (Fig. 5C-D). Multivariate analysis indicated that both CellSearch^®^ and RDW values were independent predictors of PFS and OS.

We conducted the same analysis on a cohort of patients with NSCLC (*n* = 115, stage IV), with CTCs enumerated using either the CellSearch^®^ system (*n* = 88) or Parsortix^®^ (*n* = 27). The average CTC count in this group was 3.44 (± 16.49), with only 34.78% of samples showing ≥ 1 CTC per 7.5 ml of blood. Both RDW values and CTC counts (using a threshold of ≥ 5 CTCs) were independently associated with OS (Fig. 5E and Fig. S3E, respectively). Notably, combining RDW with CTC counts ≥ 5 CTCs further enhanced the association with OS compared to RDW or CTCs alone (Fig. 5F).

In a previous study, we reported that the presence of RBCs in short-term breast cancer CTC cultures is associated with poorer outcomes [14]. Interestingly, in these patient-derived cultures, we also observed direct physical interactions between RBCs and CTCs (Fig. 5G-H).

## Discussion

Despite being a major component of blood, the role of RBCs in cancer and metastasis has been largely overlooked [2]. In this study, we investigated the interactions between tumor cells and RBCs, exploring their potential impact on metastatic processes. Our results, using tumor cell lines and RBCs from cancer patients and healthy donors (HD), demonstrated that RBCs from metastatic breast and lung cancer patients interacted more closely with cancer cells *in vitro *than RBCs from HD. To our knowledge, this is the first study to show that this interaction induces significant metabolic, morphological, and transcriptional changes in both lung and breast tumor cells, thereby enhancing their metastatic potential and malignancy.

We observed that cancer cells, when exposed to RBCs, particularly from metastatic cancer patients, undergo significant morphological changes, transitioning from a rounded shape to a more mesenchymal phenotype with lamellipodia-like protrusions. These protrusions are key membrane structures involved in chemo-sensing and migration [21] and are commonly linked to increased metastatic potential [22]. Additionally, tumor cell lines exhibited enhanced migratory and adhesive capabilities following contact with RBCs from cancer patients. Tumor cells exposed to RBCs from metastatic patients also demonstrated a marked ability to disrupt the endothelial barrier, potentially facilitating the trans-endothelial migration of CTCs into blood circulation. In vivo analyses using a zebrafish model further confirmed these findings, showing increased dissemination of tumor cells primed with RBCs from metastatic patients. Collectively, these results suggest that RBCs in metastatic cancer patients play a role in modulating tumor cells, making them more aggressive and likely contributing to tumor metastasis and progression.

Moreover, we observed extensive global effects at both the transcriptomic and proteomic levels, as demonstrated by RNA sequencing and mass spectrometry analyses, respectively. RNA-seq analysis of breast cancer cells revealed significant enrichment of adhesion-related genes and pathways involved in actin filament assembly, which aligns with our experimental findings. Among the affected genes, the upregulation of *PAK4*, *VIM*, and *PLS3*-key regulators of cytoskeletal organization, cell motility, and epithelial-to-mesenchymal transition (EMT) [23, 24, 25] - was validated by RT-qPCR analysis in MDA-MB-231 cells primed with RBCs from metastatic breast cancer patients. Furthermore, the observed increase in Plastin-3 and vimentin polarization further supports alterations in the cytoskeletal dynamics of the recipient cells [26, 27]. All three genes have been implicated in various cancers, including breast cancer, and have been correlated with poor prognosis in breast cancer patients [28, 29]. Importantly, using a specific PAK4 inhibitor (LCH-7749944) effectively reversed the increased migration and enhanced collagen I adhesion observed in MDA-MB-231 cells following exposure to RBCs from metastatic breast cancer patients. This suggests that PAK4 mediates the phenotypic and functional changes in breast cancer cells induced by RBCs. The clinical potential of PAK4 inhibitors like LCH-7,749,944 is promising, as they have demonstrated efficacy in preclinical models and are currently being evaluated in clinical trials [30, 31]. These inhibitors offer a potential therapeutic strategy aimed at mitigating the aggressive behavior of breast cancer cells influenced by RBCs from metastatic patients.

Consistent with the findings in breast cancer, mass spectrometry analyses of H1975 cells primed with RBCs from either healthy donors or cancer patients revealed significant alterations in the proteomic profile of these NSCLC tumor cells. While proteins such as vimentin and Plastin 3 showed no notable changes, other EMT markers like Keratin 5 and E-Cadherin were affected, suggesting that although similar pathways may be involved, distinct proteins play central roles in breast cancer and NSCLC. IPA revealed the enrichment of metabolic pathways and predicted increased expression of proteins related to migration, cell movement, and invasion, which is in line with our in vitro findings.

Moreover, alterations in cytoskeletal dynamics were observed, as evidenced by reduced phosphorylation (and therefore activation) of Cofilin-1, increased protein levels of gelsolin and localization of VASP at the cell protrusions after exposure to RBCs from metastatic patients. These changes suggest mechanisms by which RBC interactions may enhance actin turnover. These observations align with the work of Sidani et al., who demonstrated that modulation of Cofilin-1 expression can induce phenotypic shifts from amoeboid to mesenchymal cell types [32]. Specifically, reduced levels of Cofilin-1 were associated with decreased free F-actin barbed ends and enhanced stability of Arp2/3-mediated actin branches. This mechanistic insight highlights how interactions with RBCs from metastatic patients may trigger cytoskeletal rearrangements, leading to a more mesenchymal phenotype with increased migratory potential. Notably, our IPA analysis has already identified the activation of proteins that could be implicated in these processes. However, these findings rely primarily on expression and localization data. To further confirm the dynamic regulation of actin remodeling by RBC interactions, additional activation assays, such as RAC1 activity assessment, are required.

Our findings suggest that RBCs, within an altered systemic environment, actively promote cancer progression by modulating the aggressiveness of tumor cells in various ways. Previously, we reported modifications in the RBC proteome in patients with metastatic breast cancer [16]. Combined with the data presented here, the data indicate significant crosstalk between tumor cells and RBCs, leading to profound alterations in both entities, especially under metastatic conditions. Importantly, since mature RBCs lack nuclei and cannot synthesize new RNA, priming may occur earlier in the bone marrow through paracrine interactions, such as those involving extracellular vesicles or secreted factors [33].

Finally, patients with elevated RDW, a numerical indicator of RBC anisocytosis [34], were associated with poorer outcomes, as previously reported in meta-analyses [35], especially when CTCs were detected in the blood. Notably, RDW and CTCs serve as independent variables, each offering distinct insights into the patient’s condition: RDW reflects systemic health, while CTCs provide information about tumor burden [36, 37, 38]. Furthermore, the combined assessment of these parameters may offer a more robust prognostic biomarker. However, these compelling findings raise several important questions that warrant further investigation. Some limitations of our study should be addressed in future research before clinical application. These include examining the direct versus indirect roles of RBC interactions in larger sample cohorts, identifying key regulatory mechanisms underlying RBC-mediated effects on cancer progression, and evaluating PAK4 expression in CTCs isolated from metastatic patients to assess its prognostic potential.

## Conclusions

This study reveals a novel role for RBCs in promoting tumor cell survival and invasiveness during metastasis. By uncovering significant changes in gene expression and protein remodeling, including the upregulation of *PAK4* and alterations in actin dynamics, this research sheds light on the mechanisms driving tumor cell migration and epithelial-mesenchymal transition (EMT). Clinically, we demonstrate that integrating RBC parameters (RDW) with CTC counts enhances prognostic prediction in metastatic breast and lung cancer. These findings offer valuable new insights into the molecular mechanisms of metastasis.

## Methods

Full description of methods can be found in the supplemental information.

### Study design

The primary objective of this study was to explore the impact of RBCs interactions with tumor cells on the molecular profile and functionality of the latter. Specifically, we aimed to assess whether priming tumor cells with RBCs from HD or cancer patients (breast or lung cancer) alters their molecular characteristics or functional behavior. The tumor cells were incubated with RBCs for 24 h, followed by transcriptomic, proteomic, and functional analyses. Functional analyses focused on various stages of the metastatic cascade, including proliferation, adhesion, and migration, comparing primed and non-primed tumor cells. RNA-seq, mass spectrometry, MTT, transwell assays, adhesion assays, and electric cell-substrate impedance sensing (ECIS) assays were used. RBCs were isolated from blood samples from age-matched HD patients and cancer patients. Commercial breast and lung tumor cell lines were used in co-culture assays to evaluate potential cell line dependencies. The study design included randomising samples from sample collection stratified by tumor stage. Each experimental group (non-primed cells (HD), non-metastatic cancer patients (M0), and metastatic cancer patients (M1) with RBCs) included at least three biological replicates. Technical replicates were conducted to ensure the robustness and reproducibility of the results. Additionally, clinical data from breast (*n* = 55) and lung (*n* = 62) cancer patient cohorts were analyzed. This included CTCs enumeration and blood test data, specifically focusing on RDW values, to check the clinical relevance of the findings.

### Clinical samples

RBCs were isolated from venous blood of a cohort of both non-metastatic (M0) and metastatic (M1) breast cancer (*n* = 68) and non-small cell lung cancer (NSCLC) (*n* = 58) patients (at baseline, before treatment) and a cohort of healthy donors (HD) (*n* = 54) (Table S1-2). Samples were collected at the University Hospital Complex of Santiago de Compostela (CHUS, Spain) and the University Medical Center Hamburg-Eppendorf (UKE, Germany). Whole blood samples from patients on HD were provided by the Department of Transfusion Medicine (UKE) or volunteers (CHUS).

### Enrichment of CTCs from blood samples

The CellSearch^®^ system (Menarini) and the Parsortix^®^ microfluidic system (ANGLE) were used for CTC detection from 7.5 mL EDTA tubes of peripheral blood. For breast cancer patients, Cellsearch^®^ was used (*n* = 55), while for NSCLC cancer patients, both systems were employed (*n* = 88 and 27, respectively), following the manufacturer’s recommendations *(40*,* 41)*.

### RBC isolation and tumor cell priming

Blood samples were collected in EDTA tubes, and RBCs were isolated according to previously established protocols within two hours of collection [15]. Human cancer cell lines were co-cultured (primed) for 24 h with RBCs (2.10^6^ RBCs / 1.10^5^ tumor cells) before the start of experiments. Non-primed cell lines were used as controls (Ctrl) in each experiment.

### Cell culture

MCF-7 (ATCC HTB-22), MDA-MB-231 (ATCC CRM-HTB-26) cell lines were grown in complete DMEM supplemented with 10% Fetal Calf Serum (FCS). H1975 (ATCC CRL-5908) and A549 (ATCC CRM-CCL-185) cell lines were grown in complete RPMI-1640 supplemented with 10% FCS. HUVEC (ATCC PCS-100-013) cells were grown in EGM™-2 Endothelial Cell Growth Medium-2 BulletKit™ (Lonza), and culture plates were pretreated with 0.2% gelatin (Sigma).

### Live-cell imaging

For time-lapse recordings, RBCs and H1975 cells (1.10^6^ RBCs/3,000 tumor cells) were placed in ultra-low attachment plates and imaged using a Zeiss Apotome microscope (Carl Zeiss) in an incubator (37 °C, 5% CO_2_). A 20x objective lens was used, and images were captured every 5 min for > 18 h (*n* = 3/group).

### Electric cell-impedance sensing (ECIS)

Endothelial cell monolayer impedance was measured continuously at different frequencies (500 − 64,000 Hz) using an ECIS 1600R instrument (Applied BioPhysics, Inc.), as previously described*(42)*. HUVECs were grown to confluence, and after 24 h, half of the cell culture medium was replaced with medium containing 1.10^4^ H1975 cells (non-primed, HD, and M1 primed RBCs). A condition with medium alone, without cells, was included as a negative control. Results are expressed as relative electrical resistance with a 95% confidence interval (95% CI) (*n* = 3/group, duplicates).

### In vivo zebrafish experiments

Between 100 and 200 tumor cells were injected into the Duct of Cuvier of each fish embryo (*n* = 3/group, 33 fish injected/sample), following standard procedures*(43)*. One day post-injection, a fluorescence stereomicroscope (Nikon AZ-100) was used to image tumor spread and proliferation in caudal hematopoietic tissue. Quantifish software was used to perform image analysis *(44)*.

### RNA-Sequencing analysis and RT-qPCR

RNA was extracted using the AllPrep DNA/RNA Mini kit (Qiagen) from MDA-MB-231 cells non-primed and primed with M1 RBCs from breast cancer patients, HD RBCs, and liposomes*(45*,* 46)*, mimicking RBC membranes (*n* = 3/group, triplicates). cDNA libraries were created from 1 µg of DNase-treated RNA using the TruSeq Stranded Total RNA Globin kit (Illumina) and sequenced on an Illumina NovaSeq6000. RNA-seq data have been deposited in GEO under accession GSE273783. Gene expression was analyzed with TaqMan assays for selected genes (Table S3) and normalized to control Δct (*n* = 14/group, triplicates).

### PAK4 Inhibition

MDA-MB-231 cells were exposed for 4 h to 10–60 µM of the PAK4 inhibitor LCH-7,749,944 (Selleckchem) to determine the minimum dose of PAK4 inhibitor that affects proliferation (*n* = 3/group, quadruplicates). Proliferation was assessed at 24, 48, and 72 h using the MTT assay (Thermo Fisher) following the manufacturer´s recommendations. A concentration of 20 µM was chosen for the in vitro assays based on the IC_50_ calculations.

### Mass spectrometry

Mass spectrometry-based proteomic analyses were performed as previously described*(47)* (*n* = 5/group, four replicates). LC-MS/MS data were searched using the Sequest algorithm integrated into Proteome Discoverer software (V3.0.0.757, ThermoFisher) against a reviewed human database. Carbamidomethylation was set as a fixed modification of cysteine residues. The maximum number of two missing tryptic cleavages is set. A cutoff value (FDR < 0.01) was set for peptide and protein identification. Quantification was performed using the Minora Algorithm implemented in the Proteome Discoverer. Data were deposited in the ProteomeXchange Consortium via the PRIDE partner repository with the dataset identifier PXD053123.

### Statistical analysis

Statistical analyses were conducted using R Studio (v4.3.0) and GraphPad Prism (v8.0). Chi-square or Fisher ’s exact test was used to assess categorical variable associations. The Mann-Whitney U and Kruskal-Wallis tests were used to evaluate differences between two or more groups. Progression-free survival (PFS) and overall survival (OS) were analyzed using Kaplan-Meier plots and log-rank tests. Statistical significance was set at *P* < 0.05. RNAseq data were analyzed using DESeq2 for Fold Change and nbinomWaldTest. Gene enrichment was analyzed using ShinyGO *(48)*. Proteins in ≥ 3 samples were tested using Student’s t-test (FDR q < 0.05), and significant proteins were analyzed using IPA (Qiagen) *(49)*.

## Electronic supplementary material

Below is the link to the electronic supplementary material.

Supplementary Material 1

Supplementary Material 2

Supplementary Material 3

## Data Availability

Data are available upon request from the corresponding authors. RNA-seq data have been deposited in GEO under accession GSE273783. Mass spectrometry data were deposited in the ProteomeXchange Consortium via the PRIDE partner repository with the dataset identifier PXD053123.

## Abbreviations

CTCs: Circulating Tumor Cells
Ctrl: Control
EMT: Epithelial–Mesenchymal Transition
HD: Healthy Donor
HD RBCs: RBCs Isolated from Healthy Donors
M0: Non-Metastatic
M0 RBCs: RBCs Isolated from Non-Metastatic Patients
M1: Metastatic
M1 RBCs: RBCs Isolated from Metastatic Patients
NSCLC: Non-Small Cell Lung Cancer
OS: Overall Survival
PAKi: p21 Activated Kinase 4 Inhibitor
PFS: Progression-Free Survival
RBCs: Red Blood Cells
RDW: Red Cell Distribution Width
